# Detection of Hantavirus during the COVID-19 Pandemic, Arizona, USA, 2020

**DOI:** 10.3201/eid2908.221808

**Published:** 2023-08

**Authors:** Gavriella Hecht, Ariella P. Dale, Irene Ruberto, Guillermo Adame, Ryan Close, Sarah-Jean Snyder, Kathryn Pink, Nathanael Lemmon, Jessica Rudolfo, Michael Madsen, Andrea L. Wiens, Caitlin Cossaboom, Trevor Shoemaker, Mary J. Choi, Deborah Cannon, Inna Krapiunaya, Shannon Whitmer, Melissa Mobley, Emir Talundzic, John D. Klena, Heather Venkat

**Affiliations:** Arizona Department of Health Services, Phoenix, Arizona, USA (G. Hecht, A.P. Dale, I. Ruberto, G. Adame, H. Venkat);; Centers for Disease Control and Prevention, Atlanta, Georgia, USA (A.P. Dale, C. Cossaboom, T. Shoemaker, M.J. Choi, D. Cannon, I. Krapiunaya, S. Whitmer, M. Mobley, E. Talundzic, J.D. Klena, H. Venkat);; Indian Health Service, Whiteriver, Arizona, USA (R. Close, K. Pink, N. Lemmon);; Indian Health Service, Show Low, Arizona, USA (S.-J. Snyder);; White Mountain Apache Tribe, Whiteriver (J. Rudolfo);; Coconino County Health and Human Services, Flagstaff, Arizona, USA (M. Madsen);; Maricopa County Office of the Medical Examiner, Phoenix (A.L. Wiens)

**Keywords:** hantavirus pulmonary syndrome, viruses, COVID-19, SARS-CoV-2, respiratory infections, zoonoses, coinfection, Arizona, American Indians or Alaska Natives, United States, hantavirus Suggested citation for this article: Hecht G, Dale AP, Ruberto I, Adame G, Close R, Snyder S-J et al. Detection of hantavirus during the COVID-19 pandemic, Arizona, USA, 2020. Emerg Infect Dis. 2023 Aug [date cited]. https://doi.org/10.3201/eid2908.221808

## Abstract

We identified 2 fatal cases of persons infected with hantavirus in Arizona, USA, 2020; 1 person was co-infected with SARS-CoV-2. Delayed identification of the cause of death led to a public health investigation that lasted ≈9 months after their deaths, which complicated the identification of a vector or exposure.

The COVID-19 pandemic has affected public health investigation and response activities for other illnesses; COVID-19 has particularly challenged the diagnosis of respiratory illnesses because of similar clinical manifestations. Hantavirus pulmonary syndrome is a rare disease transmitted predominantly by infected rodents shedding the virus through saliva, urine, and feces. Sin Nombre virus is the strain of hantavirus identified in 1993 in deer mice (*Peromyscus maniculatus*) in the Four Corners region of the southwestern United States; in total, 81 human cases of hantavirus have been documented throughout Arizona through 2019 (*1*–*3*).

In March 2020, deaths of a mother and son living both on and around the White Mountain Apache Reservation in Arizona, USA, just outside of the Four Corners region, were reported to the Arizona Department of Health Services (ADHS). On September 15, 2020, the Centers for Disease Control and Prevention (CDC) notified ADHS that the mother tested positive for hantavirus, and the son was confirmed to be co-infected with both hantavirus and SARS-CoV-2. 

## The Study

**Figure 1:**
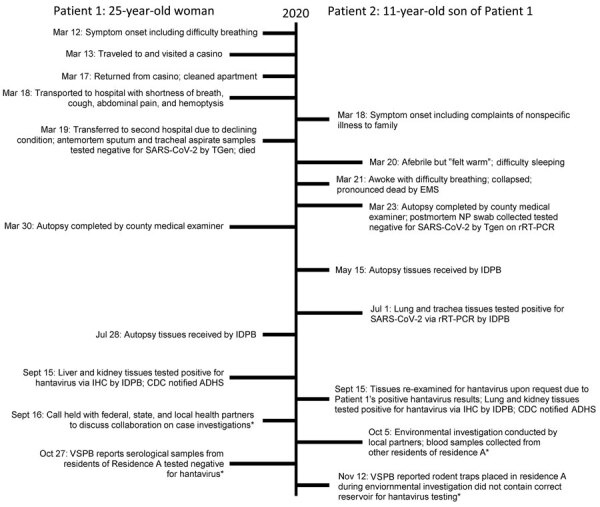
Timeline of illness course, laboratory testing, and public health investigations for 2 patients who died of hantavirus infection, Arizona, USA. Asterisk (*) indicates reported information that applies to both cases. IDPB, Infectious Diseases Pathology Branch, CDC (National Center for Emerging and Zoonotic Infectious Diseases, Division of High-Consequence Pathogens and Pathology); IHC, immunohistochemical testing; CDC, Centers for Disease Control and Prevention; NP, nasopharyngeal; rRT-PCR, real time reverse transcription PCR; TGen, Translational Genomics Research Institute; VSPB, Viral Special Pathogens Branch, CDC (National Center for Emerging and Zoonotic Infectious Diseases, Division of High-Consequence Pathogens and Pathology).

Patient 1 (P1) was a 25-year-old Native American woman with an unremarkable medical history who lived at her primary residence (residence A), a fourplex apartment on the White Mountain Apache Reservation, until her death in March 2020. She often visited her extended family at residence B, a single-family home 120 miles away, in eastern Arizona. P1 reported progressive shortness of breath beginning on March 12 (Figure 1). She stayed at a casino during March 13–17 and cleaned her apartment during March 17–18.

On March 18, P1 was transported to the hospital by emergency medical services (EMS) reporting shortness of breath, abdominal pain, and hemoptysis. In the emergency department (ED), she was febrile (temperature 101°F), tachypneic, and hypoxic; she was later intubated. Asphyxiation was initially suspected because of mixing cleaning chemicals. A chest radiograph showed diffuse bilateral infiltrates and an acute respiratory distress syndrome pattern. The ED physician documented that the radiograph looked suspicious for hantavirus, COVID-19, or diffuse bacterial pneumonia. ED physicians also diagnosed multiorgan system failure, metabolic acidosis, and metabolic encephalopathy. P1 was transferred to another hospital for a higher level of care; she was placed on extracorporeal membrane oxygenation. She died on March 19. Antemortem nasopharyngeal swab real-time reverse transcription PCR (rRT-PCR) testing for SARS-CoV-2 and respiratory viral panel testing for influenza A/B were both negative (*4*).

Patient 2 (P2) was the 11-year-old Native American male child of P1 and had an unremarkable medical history. He split time between residence A (second half of February 2020) and residence B (March 2020); he visited residence A at least 1 time in March.

On the morning of March 20, P2 was reportedly feeling unwell for 2 days and was warm but afebrile, for which he was given aspirin (dose unknown). He vomited later that day and had difficulty sleeping that evening, for which he was given 2 diphenhydramine/acetaminophen tablets (dose unknown). He awoke during the night because of difficulty breathing and collapsed out of his bed. He became unresponsive. EMS subsequently transported him to the ED, where he was pronounced dead on March 21.

The county medical examiner performed an autopsy on P2 on March 23. Postmortem nasopharyngeal swab testing for SARS-CoV-2 was rRT-PCR negative. During the autopsy, the examiner suspected an underlying pulmonary process contributing to his cause of death. Multiple tissues were sent to CDC for analysis; samples were received on May 15. On July 1, samples of P2’s lung and trachea tissues tested positive for SARS-CoV-2 by rRT-PCR (*5*), despite negative immunohistochemical results.

**Figure 2:**
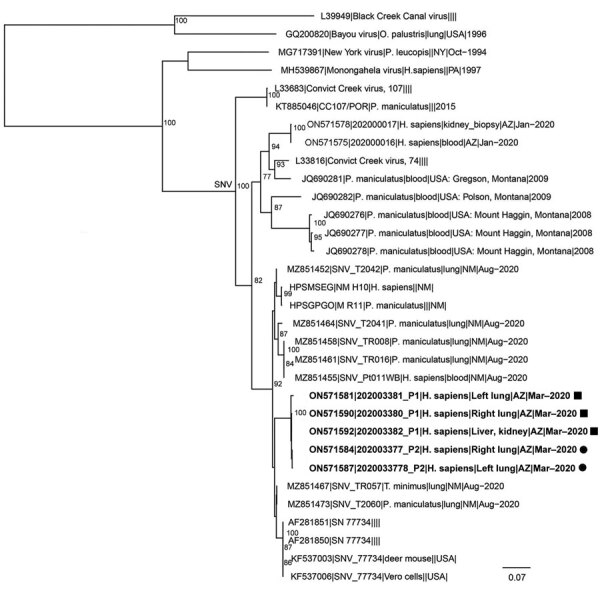
Phylogenetic tree for *Orthohantavirus* short (S) segment of samples from 2 patients who died of hantavirus infection, Arizona, USA. We inferred the phylogenetic history of full-length Sin Nombre virus S segment using maximum-likelihood estimation. Non–Sin Nombre virus species, Black Creek Canal virus, and Bayou virus are included as outgroups. Bold indicates isolates from this study; squares indicate those from patient 1 and circles those from patient 2. Numbers at nodes indicate bootstrap support >70% after 1,000 iterations. Phylogenetic trees were made using a nucleotide alignment of *Orthohantavirus* S segments. GenBank accession numbers are provided. Scale bar indicates nucleotide substitutions per site. Additional phylogenetic trees for *Orthohantavirus* medium and large segments of Sin Nombre virus are in the Appendix.

Because of the epidemiologic link between the 2 cases and unknown etiology of death for P1, tissues from P1 were submitted to CDC and received on July 28. Pathologic findings for both cases were similar; however, P1 did not have evidence of SARS-CoV-2. The pathologist observed findings that resembled hantavirus infection, which were later confirmed by positive IHC assay on liver and kidney tissues on September 15 (*5*). Because of resemblance between tissues of the 2 case-patients, P2’s tissues were reexamined, and hantavirus IHC results were positive on lung and kidney tissues. The county medical examiner later determined hantavirus to be the major contributing factor to P1 and P2’s deaths. Hantavirus genomes from P1 and P2 were closely related, indicating a common source of exposure (Figure 2). We submitted hantavirus sequences from the cases to GenBank (accession nos. ON571574–93).

On September 15, CDC alerted ADHS of the 2 positive hantavirus results. The next day, a call was held with federal, state, and local partners to coordinate a collaborative case investigation. On October 5, the Indian Health Service (IHS) conducted an environmental investigation of residence A and collected 16 human blood samples from 17 residents living in the fourplex, including household members of P1 and P2. Hantavirus serology assays for 16 human samples all tested negative for hantavirus IgM and IgG (*6*). IHS provided hantavirus prevention public service announcements to local health officials; the announcements were later disseminated to the community through newspaper and radio.

We conducted an environmental investigation because both residence A and residence B displayed potential for deer mice habitat. Trapping conducted in residence A confirmed the presence of rodents by identifying house mice (*Mus musculus*) in 4 of 6 snap traps; we did not test the mice because that species is not a known reservoir for hantavirus. Unfortunately, we were not able to conduct trapping at residence B. All partners involved mutually decided to end the investigation.

The time interval from symptom onset to diagnosis was ≈6 months. Despite the local ED physician suspecting hantavirus in P1, medical records showed no evidence of hantavirus testing ordered at either hospital. Local health and medical staff were focused on the response to initial cases of SARS-CoV-2 in the region. The time between postmortem tissue submission and subsequent sample testing contributed to the delay. Testing delays might have resulted from CDC requirement of confirmatory diagnostic testing on all confirmed or suspected COVID-19 deaths (*5*). Hantavirus was identified 6 weeks after P2’s tissue examination; P1’s lung tissue resembled that of P2 but tested negative for SARS-CoV-2. Delays in case identification caused the environmental investigation to be conducted 7 months after disease onset. The rodent population might have changed during that period, preventing identification of the vector and exposure.

## Conclusions

In the cases we report, hantavirus infection was not promptly detected when patients sought medical care or during postmortem examination. To aid in the diagnosis of hantavirus, we recommend adoption of the 5-point hantavirus screening tool for areas outside the Four Corners region (*7*,*8*). The COVID-19 pandemic likely played a role in delayed detection of hantavirus for these cases because of its effects on aspects of healthcare and public health (*9*–*12*). Hantavirus education should continue to be a priority in healthcare facilities in disease-endemic regions, including on tribal lands. Community education can help to minimize the impact of hantavirus cases by offering tools to prevent exposure and encourage seeking prompt medical care.

During pandemic response, public health partners should continue to monitor and respond to other pathogens. Medical providers should consider both alternative and concurrent diagnoses in the presence of COVID-19–like illness, including rare pathogens such as hantavirus. Timely investigations of high-consequence illnesses will enable public health organizations to take prompt action.

Appendix Additional information about hantavirus infection in 2 patients during the COVID-19 pandemic, Arizona, 2020.
