# Supplementary material for: Detection of Hantavirus during the COVID-19 Pandemic, Arizona, USA, 2020
Source: Emerg Infect Dis. 2023 Aug;29(8):1663–7. doi: 10.3201/eid2908.221808 (PMC10370831; doi:10.3201/eid2908.221808)
Supplement: Appendix — Additional information about hantavirus infection in 2 patients during the COVID-19 pandemic, Arizona, 2020. [file 22-1808-Techapp-s1.pdf]

Article DOI: http://doi.org/10.3201/eid2908.221808

EID cannot ensure accessibility for supplementary materials supplied by authors. Readers who have difficulty accessing supplementary content should contact the authors for assistance.

Detection of Hantavirus During the COVID-19 Pandemic, Arizona, 2020

Appendix

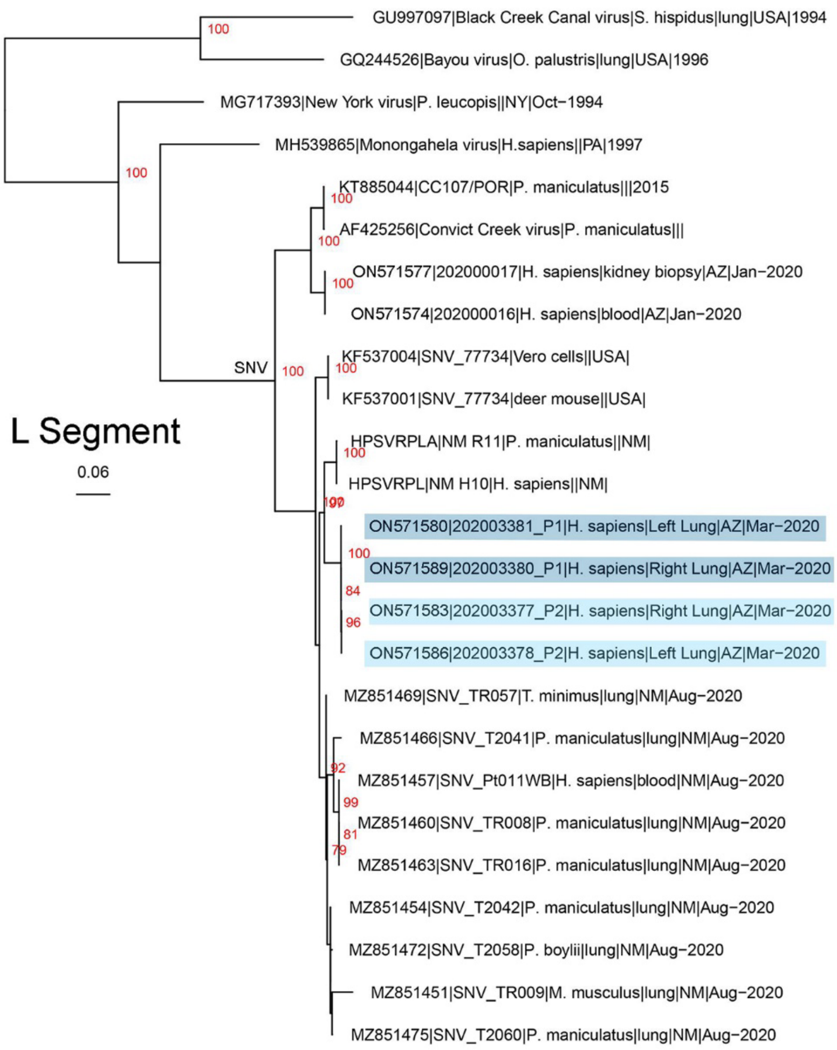

Appendix Figure 1. Phylogenetic tree for *Orthohantavirus* long (L) segment. We inferred phylogenetic history of full-length SNV L segment using maximum-likelihood estimation. Phylogenetic trees were made using a nucleotide alignment of *Orthohantavirus* L segments.

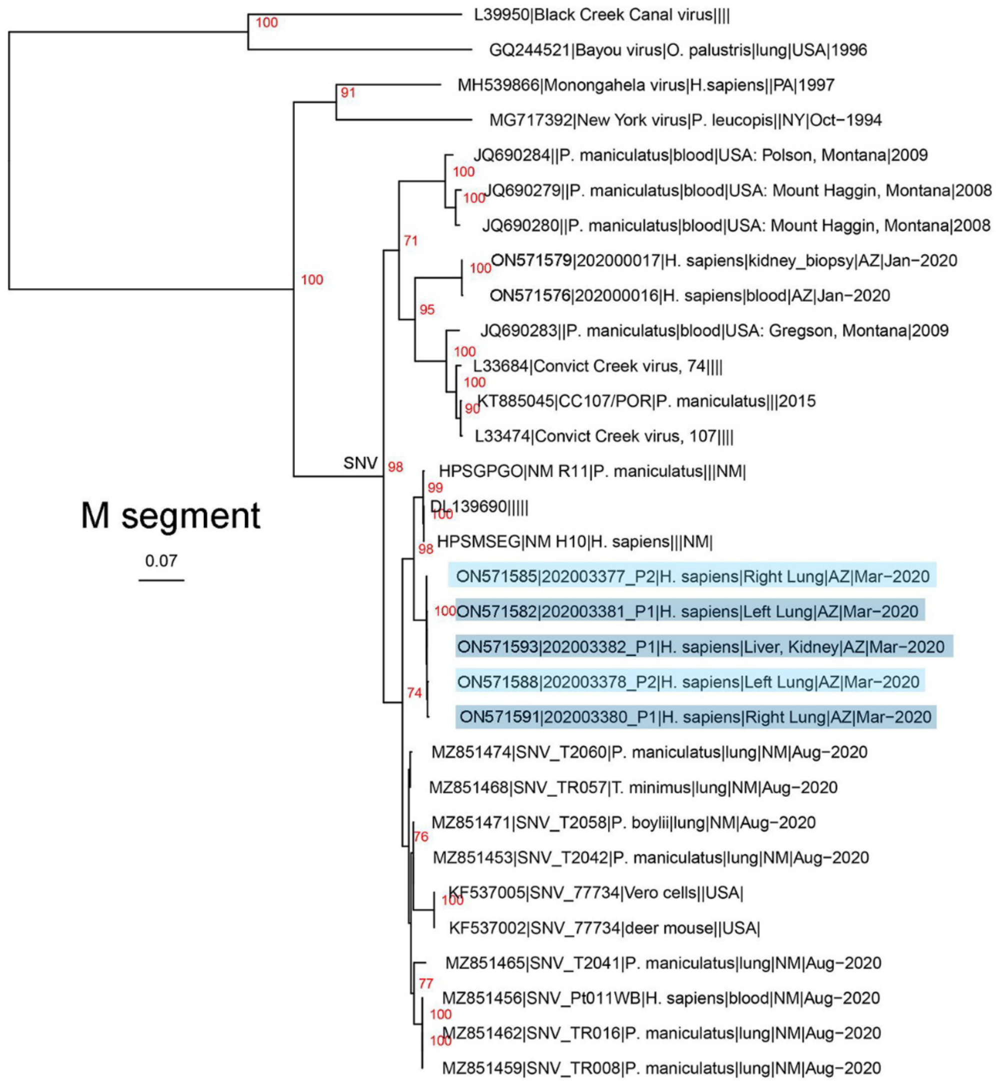

Appendix Figure 2. Phylogenetic tree for *Orthohantavirus* medium (M) segment. We inferred phylogenetic history of full-length SNV M segment using maximum-likelihood estimation. Phylogenetic trees were made using a nucleotide alignment of *Orthohantavirus* M segments.
